# Time-of-flight secondary ion mass spectrometry fragment regularity in gallium-doped zinc oxide thin films

**DOI:** 10.1038/s41598-021-87386-6

**Published:** 2021-04-07

**Authors:** K. G. Saw, S. R. Esa

**Affiliations:** 1 School of Distance Education, Universiti Sains Malaysia, 11800 Penang, Malaysia; 2 MIMOS Semiconductor (M) Sdn Bhd, Technology Park Malaysia, 57000 Kuala Lumpur, Malaysia

**Keywords:** Materials science, Physics

## Abstract

Time-of-flight secondary ion mass spectrometry fragment analysis remains a challenging task. The fragment appearance regularity (FAR) rule is particularly useful for two-element compounds such as ZnO. Ion fragments appearing in the form of Zn_*x*_O_*y*_ obey the rule $2x \ge 2y + 1$ in the positive secondary ion spectrum and $2x \le 2y + 1$ in the negative spectrum where the valence of Zn is + 2 and that of O is − 2. Fragment analysis in gallium-doped ZnO (GZO) films can give insights into the bonding of the elements in this important semiconductor. Fragment analysis of 1 and 7 wt% GZO films shows that only the negative ion fragments obey the FAR rule where ZnO^‒^, ^66^ZnO^‒^, ^68^ZnO^‒^ and ZnO_2_^‒^ ion fragments appear. In the positive polarity, subdued peaks from out-of-the-rule ZnO^+^, ^66^ZnO^+^ and ^68^ZnO^+^ ion fragments are observed. The Ga ion peaks are present in both the positive and negative spectra. The secondary ion spectra of undoped ZnO also shows consistency with the FAR rule. This implies that Ga doping even in amounts that exceed the ZnO lattice limit of solubility does not affect the compliance with the FAR rule.

## Introduction

Time-of-flight secondary ion mass spectrometry (ToF–SIMS) fragment analysis is an elaborate and challenging task. Among the analysis methods such as multivariate analysis and principal component analysis, the fragment appearance regularity (FAR) rule is particularly useful for two-element compounds where the valence and electronegativity of the elements are known^1–5^. The FAR rule originates from a study on Ga^+^ primary ion fragment patterns of inorganic compounds and metals. It suggests that for a two-element compound MA, ion fragments appearing in the form of M_x_A_y_ will obey the rule $nx \ge py + 1$ in the positive spectrum and $nx \le py + 1$ in the negative spectrum^4,5^. Here, the valence of cation M is + *n* and that of anion A is − *p*, while the electronegativity of M is smaller than that of A. The electronegativity and valence of an atom are therefore two important factors in the generation of positive and negative ion fragments. In metal oxides, the FAR rule assumes that the valence of O remains stable as − 2^4^. For instance, in the fragment analysis of CuO, the valence of O is maintained as − 2 while the valence of Cu is + 2. Within-the-rule ion fragments Cu_3_O^+^ and Cu_4_O_2_^+^ are observed in the positive secondary ion spectrum as inferred. In the negative spectrum, however, Cu_2_O^‒^ and Cu_3_O_2_^‒^ appear due to reduction of the valence state of Cu caused by the irradiation of the primary ion beam^4,5^. This implies that *n* is now + 1 instead of + 2 and the rule $nx \le py + 1$ is still obeyed. Previous studies have indicated that the FAR rule was observed for the oxides of some metals such as Al, Si and Ni^4–7^. In the investigation of NiO fragmentation behaviour, Ni is assumed to have a valence of + 2. The Ni_3_O^+^, NiO^‒^, NiO_2_^‒^, Ni_2_O_2_^‒^, Ni_2_O_3_^‒^, Ni_2_O_5_^‒^, Ni_3_O_3_^‒^, Ni_3_O_4_^‒^ and Ni_4_O_4_^‒^ ion fragments are thus within the rule^5^.

It is interesting to note within-the-rule ion fragments are not limited to Ga^+^ primary ions but are also generated by Ar^+^ primary ions^8^. For instance, in the investigation of Nb_2_O_3_ catalysts supported on TiO_2_, the reported negative ion fragments NbO_2_^‒^, NbO_3_^‒^, Nb_2_O_5_^‒^, Nb_2_O_6_^‒^, Nb_3_O_7_^‒^, and Nb_4_O_10_^‒^ are within the rule^8^. This shows that compliance with the FAR rule is not affected by the monatomic primary ion species. Secondary ion spectra generated by different monatomic primary ions were found to be similar in a recent study on PCI analysis on protein^1^. With advances in primary ion technology, primary ions such as B_n_^q+^ (n = 2 and 3, q = 1 and 2) and C_60_^q+^ (q = 1–3) are increasingly used for better yield of ionized fragments particularly in certain organic and biological samples^1^ although Bi_1_^+^ is widely used for inorganic materials such as metal oxides. Some insights on the fragmentation behaviour can be obtained from recent molecular dynamic (MD) simulations into the implantation and probing depths of primary ion species during bombardment^1^. The MD simulations indicate that the use of electropositive ion species (such as Ga^+^ and Bi_1_^+^) can cause the excitation of more secondary electrons over the surface potential barriers. The increased number of electrons subsequently leads to enhanced negative secondary ion formation especially for elements with high electron affinity.

There has been an apparent lack of research in the FAR rule on doped metal oxides. The fragmentation behaviour of a doped metal oxide such as ZnO that exhibits metal-like conductivity and bandgap widening when doped with a small wt% of metal dopants is an interesting research area. No study on the FAR rule for ZnO (doped as well as undoped) has been reported to the best of our knowledge. For ZnO, the valence of Zn is + 2 while that of O is − 2. Ion fragments appearing in the form of Zn_x_O_y_ thus obey $2x \ge 2y + 1$ in the positive spectrum and $2x \le 2y + 1$ in the negative spectrum. The electronegativity of Zn is 1.65, which is comparatively smaller than that of O (3.44), and therefore satisfies the requirement of the FAR rule. ZnO has a hexagonal wurtzite structure and a wide bandgap of approximately 3.3 eV with potential applications in industrial catalysts, optoelectronic devices and green technology such as waste management^9–19^. Undoped ZnO is usually too resistive for transparent conducting oxide applications and requires donor dopants such as Ga on Zn sites^12^. In polymer solar cells, gallium-doped ZnO (GZO) is used as a cathode interfacial layer to enhance photovoltaic performance^18^. Recently, magnetron sputtered GZO thin films with a thickness of 361 nm have been used together with CuI to build a transparent *p*-*n* thermoelectric module interconnected with indium tin oxide as a transparent electrode^19^. Magnetron sputtering remains a popular synthesis technique because deposition of high-purity GZO thin films can be achieved on a large scale at relatively low temperatures^20–23^. Magnetron sputtered GZO films synthesized at elevated substrate temperatures up to 200 °C are usually crystalline and highly *c*-axis oriented. Higher substrate temperatures, however, may cause the unwanted formation of ZnGa_2_O_4_ and result in films with poorer crystalline quality^23^. Other techniques for synthesizing GZO films include spray pyrolysis^24^, sol–gel method^25^, sonochemical assisted method^26^, spin-coating^17^, pulsed laser deposition^27^ and chemical vapour deposition^12,28^. Ga doping levels in ZnO usually range between 1 and 7 wt%^29–31^ as there is evidence that doping beyond 6.68 wt% causes the ZnO *c*-axis growth orientation to deteriorate and the UV emission to be significantly reduced^31^.

In this work, we investigate the fragment patterns of GZO films with different amounts of Ga dopants for compliance with the FAR rule. Fragment analysis using ToF–SIMS data from our previous work on undoped ZnO^32^ shows that only the negative secondary ion spectrum obeys the FAR rule with the appearance of ZnO^–^, ^66^ZnO^–^, ^68^ZnO^–^ and ZnO_2_^–^ peaks. Out-of-the-rule ZnO^+^ and ^66^ZnO^+^ ion fragments are observed in the positive polarity. The positive and negative secondary ion spectra of undoped ZnO are shown in Supplementary Figs. S1 and S2, respectively. The ion fragments are listed in Supplementary Tables S1 and S2. X-ray photoelectron spectroscopy (XPS) and field emission scanning electron microscopy (FE-SEM) analyses indicate a continuous ZnO film (Supplementary Fig. S3). The at% of Zn and O are shown in Supplementary Table S3. The ToF–SIMS data as well as preparation details and other characterizations were reported in our previous study^32^. To extend our previous work on undoped ZnO, this present study investigates the effect of different levels of Ga doping on the fragment patterns. Two sputtering targets with different amounts of Ga (99 wt% ZnO:1 wt% Ga_2_O_3_ and 93 wt% ZnO:7 wt% Ga_2_O_3_) are used. The films from these two targets are labelled as 1 and 7 wt% GZO films.

This paper begins with an explanation of the FAR rule supported by various studies on metal oxides. The results of the present investigation are next presented and the compliance of the ion fragments of GZO is discussed. The conclusion is then drawn. The paper ends with a section on materials and methods used in this study.

## Results and discussion

**Figure 1:**
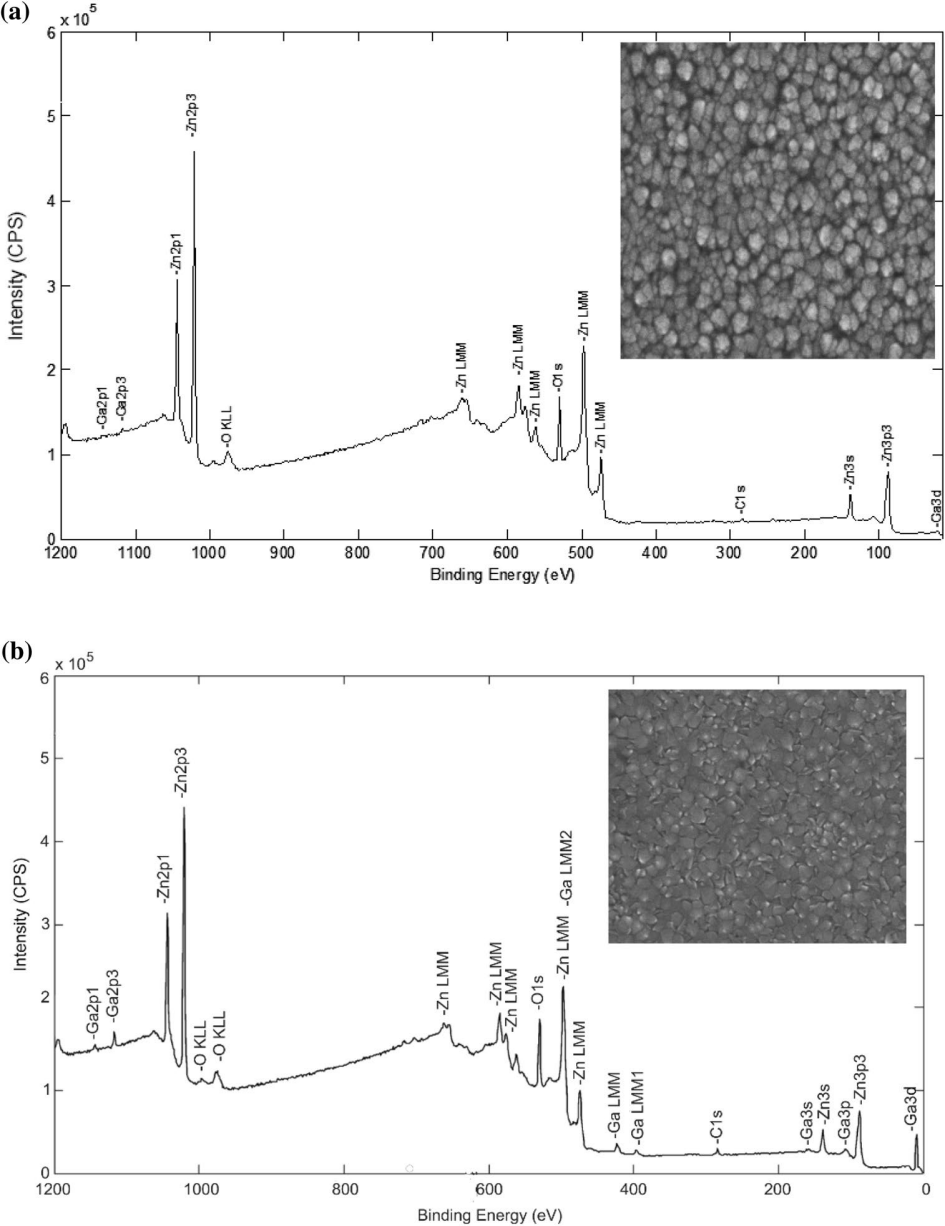
XPS survey spectrum of the (**a**) 1 wt% and (**b**) 7 wt% GZO film. Inset is the FE-SEM image showing a continuous surface morphology (mag. ×50000).

Surface composition analysis using XPS survey scan shows the Zn 2*p*, O 1*s* and Ga 2*p* peaks, confirming the existence of the corresponding elements in the GZO films (Fig. 1a,b). Except for the C 1*s* peak attributed to adventitious carbon, no other elements were detected in the films. The wt% of Ga, Zn and O of the GZO films are shown in Supplementary Table S4. The corresponding field-emission scanning electron microscope (FE-SEM) images of the continuous surface morphology of the 1 and 7 wt% films are shown as insets in Fig. 1a,b, respectively.

The XRD 2-theta spectra of the 1 and 7 wt% GZO films synthesized at 150 °C (Supplementary Fig. S4) show intense diffraction peaks indexed as the hexagonal wurtzite phase of ZnO. The diffraction peaks are located at 34.31° and 34.23° for the 1 and 7 wt% GZO films, respectively. The corresponding full-width at half-maximum (FWHM) values are 0.15° and 0.37°. A *c*-axis preferential growth direction perpendicular to the substrate plane is thus implied. The (002) peak of the 1 wt% GZO film deposited at room temperature is observed at 34.27° with a larger FWHM value of 0.25°. A higher deposition temperature of 150 °C is thus used for better crystalline quality. The FWHM value of the 7 wt% GZO film indicates that a higher amount of Ga doping decreases the crystalline quality. The hexagonal wurtzite structure of the GZO films is confirmed by the E_2_ (H) peak at 438 cm^-1^ in the Raman spectrum (Supplementary Fig. S5). The Raman active zone center optical phonons are A_1_ + E_1_ + 2E_2_ + 2B_1_ where A_1_ and E_1_ are polar modes while the E_2_ modes are non-polar with two frequencies. The E_2_ (H) and E_2_ (L) are associated with oxygen displacement and Zn sub-lattice respectively. The B_1_ are silent modes. The peaks labelled 1 and 2 are from the underlying Si substrate.

**Figure 2:**
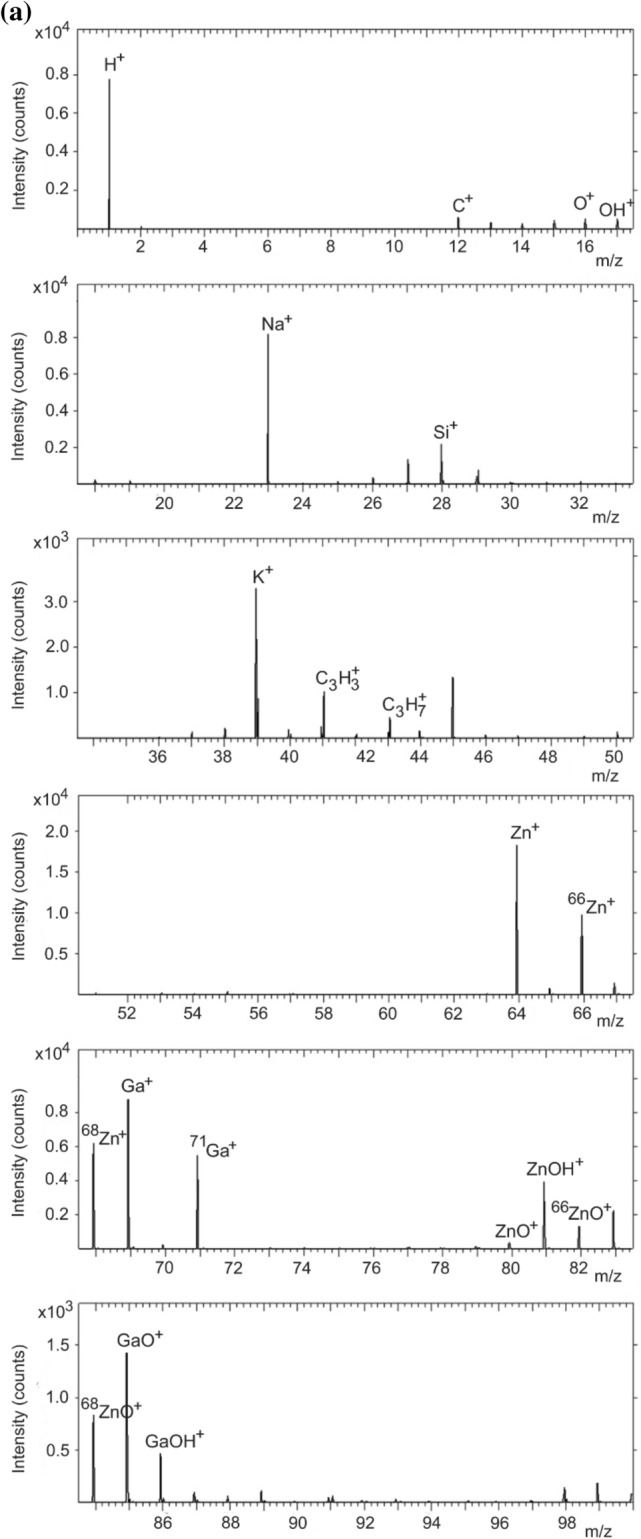

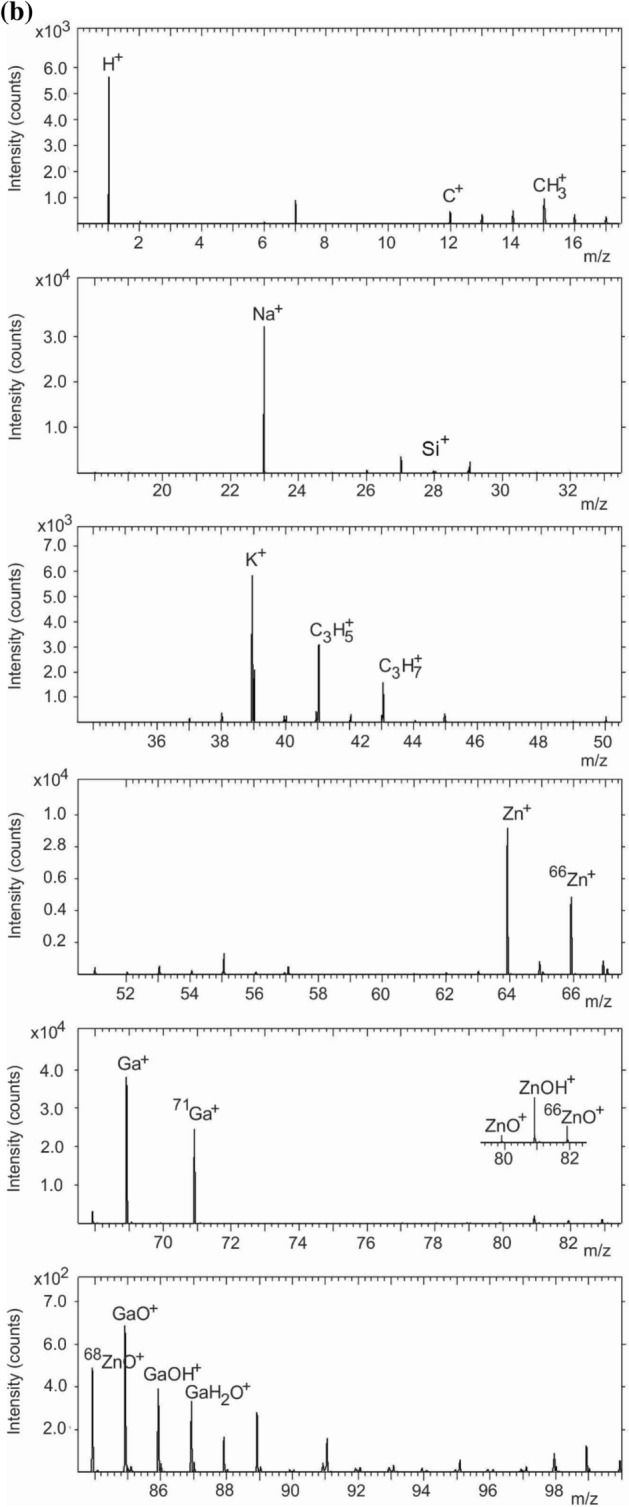
Positive secondary ion spectrum for the (**a**) 1 wt% and (**b**) 7 wt% GZO film.

In the fragment analysis of ZnO, the FAR rule suggests that the valence of O is − 2 while that of Zn is + 2. The rule $2x \ge 2y + 1$ thus applies to positive ion fragments while negative fragments obey the rule $2x \le 2y + 1$. Figure 2a,b show the positive secondary ion spectra obtained from the 1 and 7 wt% GZO films, respectively. Ion fragments from the 1 wt% GZO film include ZnO^+^ (*m/z* 79.922), ^66^ZnO^+^ (*m/z* 81.919), ^68^ZnO^+^ (*m/z* 83.933) as well as H^+^ (*m/z* 1.007), Na^+^ (*m/z* 22.989), K^+^ (*m/z* 38.963), Si^+^ (*m/z* 27.976), C^+^ (*m/z* 12.000), C_3_H_5_^+^ (*m/z* 41.039), C_3_H_7_^+^ (*m/z* 43.056), Zn^+^ (*m/z* 63.928), ^66^Zn^+^ (*m/z* 65.925), ^68^Zn^+^ (*m/z* 67.923), ZnOH^+^ (*m/z* 80.930), Ga^+^ (*m/z* 68.924), ^71^ Ga^+^ (*m/z* 70.923), GaO^+^ (*m/z* 84.926) and GaOH^+^ (*m/z* 85.933). Ion fragments from the 7 wt% GZO film include ZnO^+^ (*m/z* 79.922), ^66^ZnO^+^ (*m/z* 81.919), ^68^ZnO^+^ (*m/z* 83.934) as well as H^+^ (*m/z* 1.007), Na^+^ (*m/z* 22.990), K^+^ (*m/z* 38.963), Si^+^ (*m/z* 27.976), C^+^ (*m/z* 12.000), C_3_H_5_^+^ (*m/z* 41.040), C_3_H_7_^+^ (*m/z* 43.056), Zn^+^ (*m/z* 63.928), ^66^Zn^+^ (*m/z* 65.925), ZnOH^+^ (*m/z* 80.931), Ga^+^ (*m/z* 68.925), ^71^ Ga^+^ (*m/z* 70.924), GaO^+^(*m/z* 84.926), GaOH^+^(*m/z* 85.931) and GaH_2_O^+^ (*m/z* 86.934).

In Fig. 2a, the ^68^ZnO^+^, ZnO^+^ and ^66^ZnO^+^ peaks are observed. The Ga-containing fragments are Ga^+^, ^71^ Ga^+^, GaO^+^ and GaOH^+^. Ga has two naturally occurring isotopes with ^69^ Ga as the predominant isotope (60.11%) and ^71^ Ga with an abundance of 39.89%. Unlike CuO, the appearance of ion fragments not inferred by the FAR rule where *n* = 2 and *p* = 2 cannot be attributed to a change in the valence of Zn. This would imply an increase of *n* to 3 which is unlikely for Zn. Thus ZnO^+^, ^66^ZnO^+^ and ^68^ZnO^+^ ion fragments in the 1 wt% GZO film are considered as out-of-the-rule. The positive secondary ion spectrum from the 7 wt% GZO film (Fig. 2b) also shows consistency with the FAR rule.

Hydrogen usually forms positive as well as negative secondary ions as it is present in the residual gas in the ToF–SIMS instrument even at a base pressure of 10^‒10^ Torr^33^. It is also easily adsorbed onto the sample surface during the primary ion bombardment. The ion fragment H^+^ appears as an intense peak (Fig. 2a,b) together with Na^+^, K^+^, Si^+^, C_3_H_5_^+^ and C_3_H_7_^+^ that are from adsorbed atmospheric particulates^34–36^. The Na^+^ and K^+^ alkali metal contaminants are easily ionized (and hence their intense peaks) because their ionization potentials are comparable with the ionization potentials of the primary ion that was used^5^. In particular, Na compounds in the atmosphere tend to accumulate easily on the surfaces of aerosol particles when the relative humidity is high^36^. The ion fragments of both positive secondary ion spectra are listed in Supplementary Tables S5 and S6.

**Figure 3:**
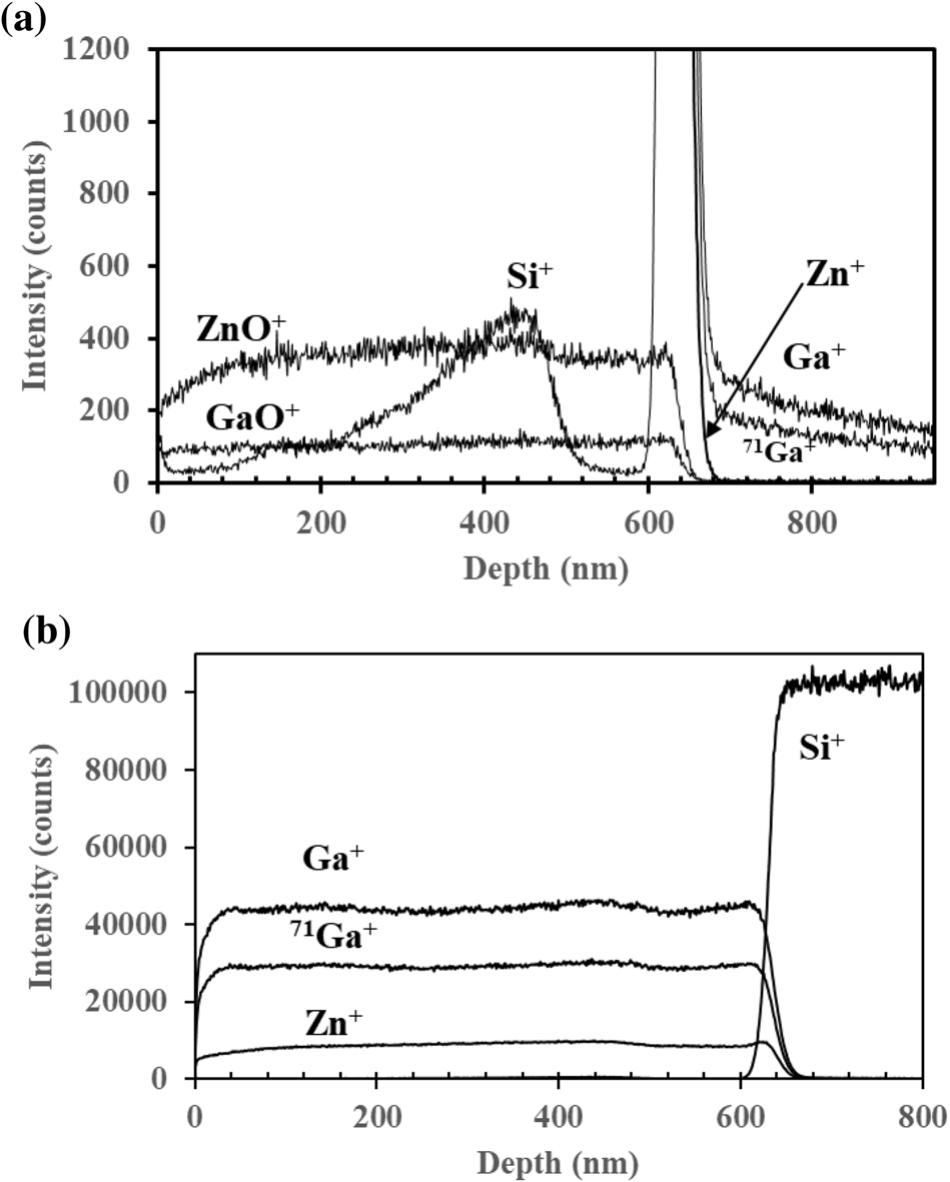
Depth profile analysis of (**a**) low intensity Si^+^, ZnO^+^, GaO^+^ ions and (**b**) high intensity Zn^+^, Ga^+^ and ^71^ Ga^+^ ions.

The depth profiles of the Si^+^, ^68^ZnO^+^ and GaO^+^ of the 7 wt% GZO film are depicted in Fig. 3a. Their intensities are much lower compared to the Zn^+^, Ga^+^ and ^71^ Ga^+^ ions, which appear as intense peaks in the positive polarity. The low intensity of Si^+^ ion suggests that Si that is detected in the film originates from atmospheric contaminants. The out-of-the rule ZnO^+^ is detected throughout the GZO film. The high intensity shown by the depth profiles of Zn^+^, Ga^+^ and ^71^ Ga^+^ ion in Fig. 3b throughout the film suggests the elemental Zn ion fragments (i.e. Zn^+^, ^66^Zn^+^, and ^68^Zn^+^) originate from the ZnO film rather than atmospheric contaminants. The depth profile analysis also confirms the homogeneity of Ga throughout the ZnO thin films.

**Figure 4:**
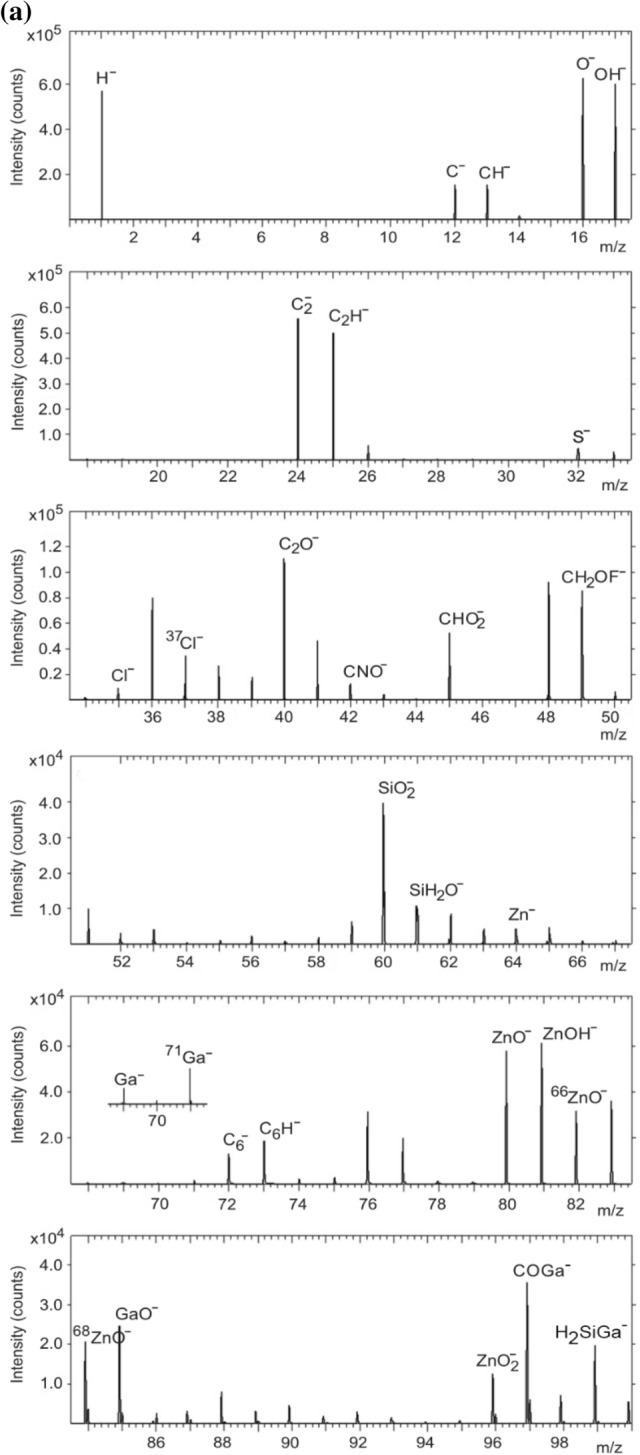

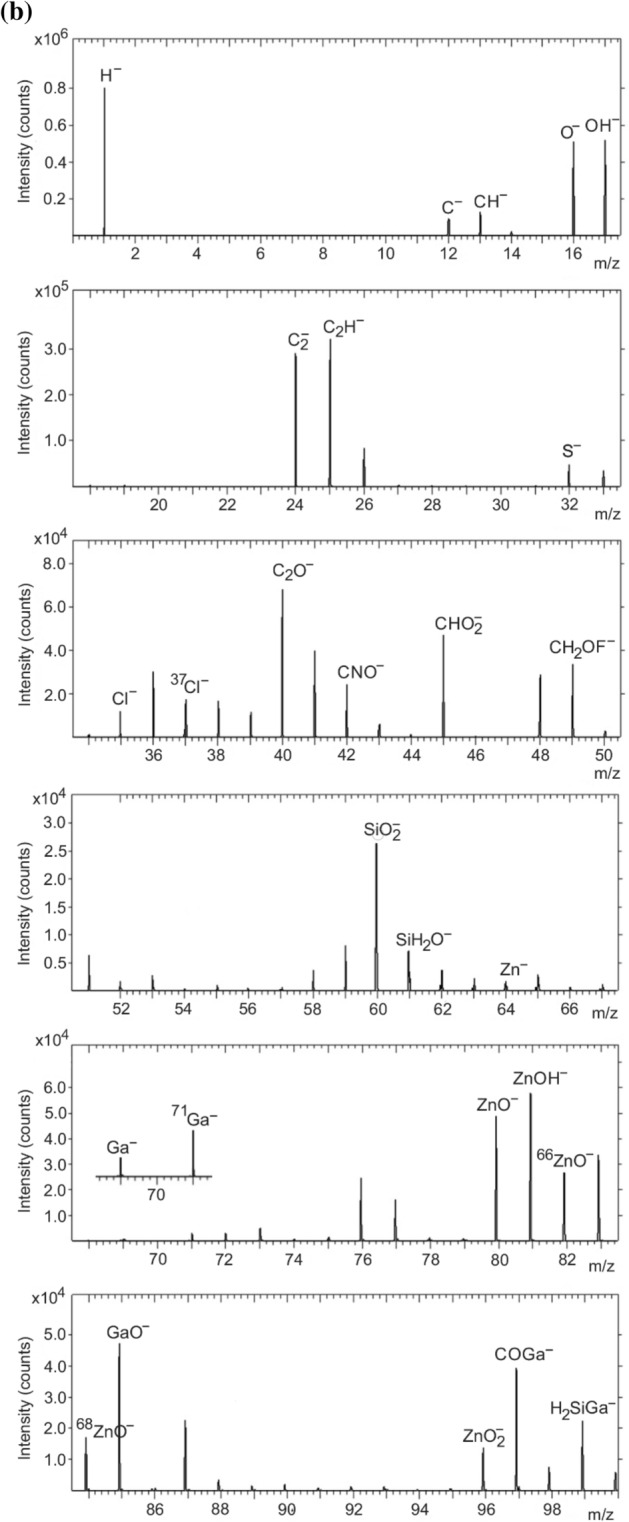
Negative secondary ion spectrum for the (**a**) 1 wt% and (**b**) 7 wt% GZO film.

Figure 4a,b show the negative secondary ion spectra obtained from the 1 and 7 wt% GZO films, respectively. There is evidence of compliance with the FAR rule in both films. In the 1 wt% film (Fig. 4a), peaks originating from within-the-rule fragments ZnO^‒^, ^66^ZnO^‒^, ^68^ZnO^‒^ and ZnO_2_^‒^ appear at *m/z* 79.918, *m/z* 81.913, *m/z* 83.913 and *m/z* 95.910, respectively. The other peaks are assigned to ZnOH^‒^ (*m/z* 80.926), GaO^‒^ (*m/z* 84.919), COGa^‒^ (*m/z* 96.919), H_2_SiGa^‒^ (m/z 98.915) as well as H^‒^ (*m/z* 1.010), O^–^ (*m/z* 15.998), OH^–^ (*m/z* 17.006), C_2_^‒^ (*m/z* 24.004), C_2_H^‒^ (*m/z* 25.002), C_2_O^‒^ (*m/z* 39.996), CHO_2_^‒^ (*m/z* 44.998) and CH_2_OF^‒^ (*m/z* 49.010). In the 7 wt% film (Fig. 4b), within-the-rule fragments ZnO^‒^, ^66^ZnO^‒^, ^68^ZnO^‒^ and ZnO_2_^‒^ appear at *m/z* 79.922, *m/z* 81.918, *m/z* 83.919 and *m/z* 95.917, respectively. The other peaks are assigned to ZnOH^‒^ (*m/z* 80.931), GaO^‒^ (*m/z* 84.922), COGa^‒^ (*m/z* 96.926), H_2_SiGa^‒^ (m/z 98.922) as well as H^‒^ (*m/z* 1.009), O^–^ (*m/z* 15.997), OH^–^ (*m/z* 17.005), C_2_^‒^ (*m/z* 24.003), C_2_H^‒^ (*m/z* 25.011), C_2_O^‒^ (*m/z* 39.996), CHO_2_^‒^ (*m/z* 44.999) and CH_2_OF^‒^ (*m/z* 49.010).

Unlike the positive secondary spectrum, the Ga^‒^ (*m/z* 68.926) and ^71^ Ga^‒^ (*m/z* 70.924) ion fragments appear with lower intensity in the negative polarity. Ga therefore forms both positive and negative secondary ions. The sulfur ion fragment originates from atmospheric particulates^33^ (Fig. 4a,b). In both the 1 and 7 wt% films, elemental Si ion fragments as well as fragments containing Si result in intense peaks of SiO_2_^‒^ and SiH_2_O^‒^. The C^‒^, CH^‒^, C_2_^‒^, C_2_H^‒^, Cl^‒^, ^37^Cl^‒^, C_2_O^‒^, CNO^‒^, CHO_2_^‒^, and CH_2_OF^‒^ ion fragments (Fig. 4a,b) are also from atmospheric contaminants. Previous studies on surfaces of atmospheric particulates (e.g. PM_2.5_) have detected secondary ions such as Li^+^, F^‒^, O^‒^, Na^+^, Mg^+^, Al^+^, Si^+^, NH_3_^+^, NH_4_^+^, C_3_H_3_^+^, C_7_H_7_^+^, C_2_H^‒^, NO^‒^, NO_2_^‒^, CN^‒^, CNS^‒^, O_2_^‒^, HS^‒^, PO_2_^‒^, SO^‒^, SO_2_^‒^, SO_3_^‒^, SO_4_^‒^ and HSO_4_^‒^^34–36^.

**Figure 5:**
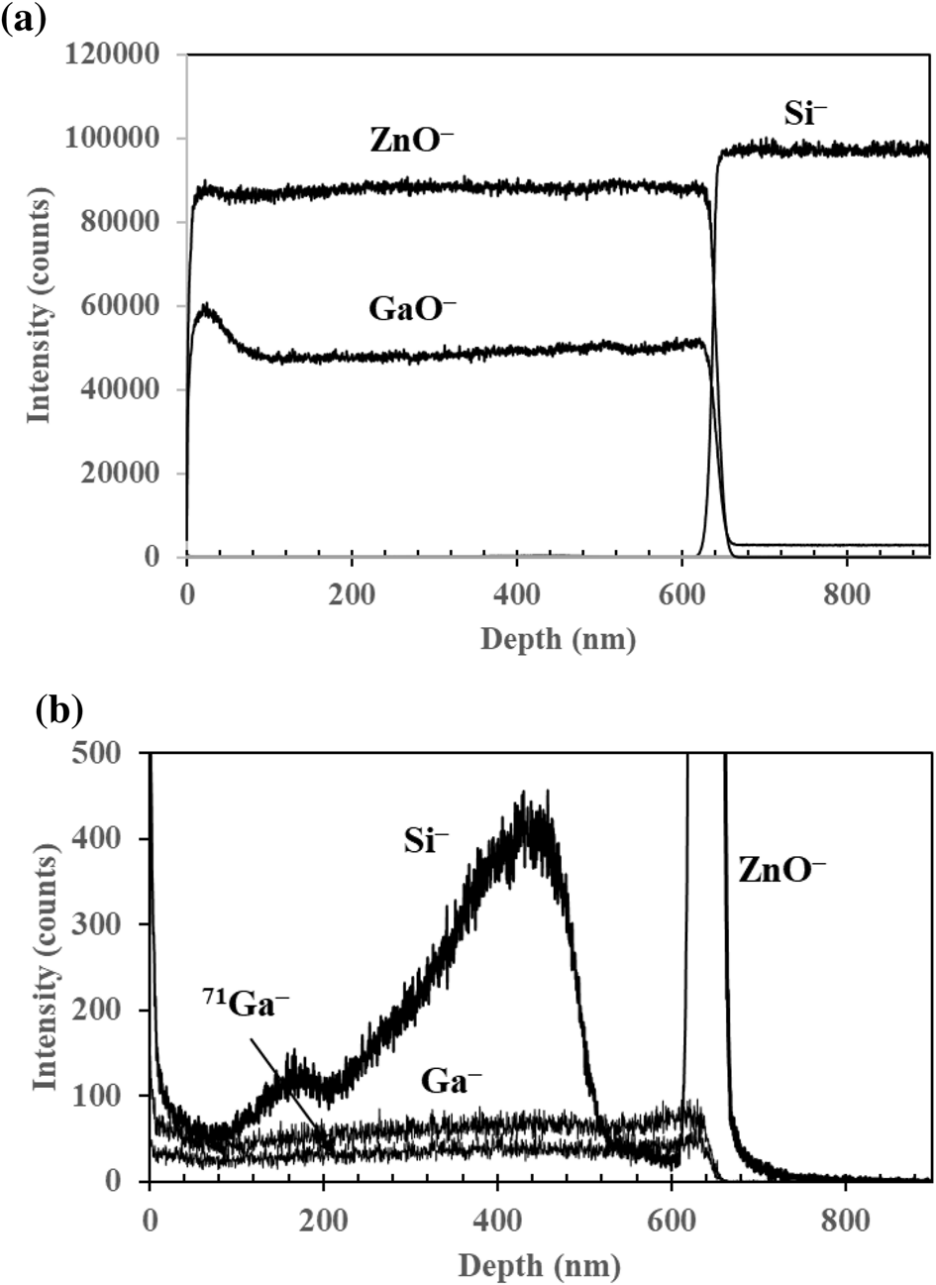
Depth profile analysis of (**a**) high intensity ZnO^‒^, GaO^‒^ and Si^‒^ ion fragments and (**b**) low intensity Si^‒^, Ga^‒^ and ^71^ Ga^‒^ ion fragments.

Figure 5a shows the depth profile analysis of ZnO^‒^, GaO^‒^ and Si^‒^ from the 7 wt% GZO film. The ion yields of ZnO^‒^ and GaO^‒^ decrease abruptly as expected at the interface between the film and the underlying Si substrate but Si^‒^ experiences a sharp increase as depth profiling continues into the Si substrate. The depth profile of Si^‒^ originating from atmospheric contaminants in the GZO film is not seen in Fig. 5a due to the high intensity of the ZnO^‒^ and GaO^‒^ ions. Depth profiles of the low intensity Si^‒^, Ga^‒^ and ^71^ Ga^‒^ ions are depicted in Fig. 5b. It is therefore reasonable to conclude that the SiO^‒^ and SiHO^‒^ ion fragments observed in the negative secondary ion spectra originate from atmospheric contaminants. The source of Si^‒^ beyond the thickness of the film is the underlying Si substrate. The ion fragments of both negative secondary ion spectra are listed in Supplementary Tables S7 and S8.

From our previous study on the undoped ZnO film^32^, out-of-the-rule ZnO^+^ and ^66^ZnO^+^ fragments are detected at *m/z* 79.924 and *m/z* 81.922, respectively (Supplementary Fig. S1). Intense peaks assigned to elemental ions ^64^Zn^+^ and ^66^Zn^+^ ions are observed at *m/z* 63.930 and *m/z* 65.927, respectively. Similarly in the negative secondary ion spectrum (Supplementary Fig. S2), within-the-rule ZnO^–^, ^66^ZnO^‒^, ^68^ZnO^‒^ and ZnO_2_^‒^ ion fragments appear at *m/z* 79.924, *m/z* 81.921, *m/z* 83.922, and *m/z* 95.920 respectively. These results are consistent with the results of the present work on GZO films in relation to the FAR rule.

The incorporation of Ga into ZnO causes the Ga atom to be ionized into Ga^3+^ which then replaces the Zn^2+^ ion in the ZnO host lattice. This substitution contributes one free electron and thus increases the carrier concentration according to Eq. (1).

1 $${\text{Ga}}_{2} {\text{O}}_{3} \to 2{\text{Ga}}_{{{\text{Zn}}}}^{ \cdot } + 2{\text{O}}_{{\text{O}}}^{{\text{X}}} + 2e^{\prime} + \frac{1}{2}{\text{O}}_{2}$$

**Figure 6:**
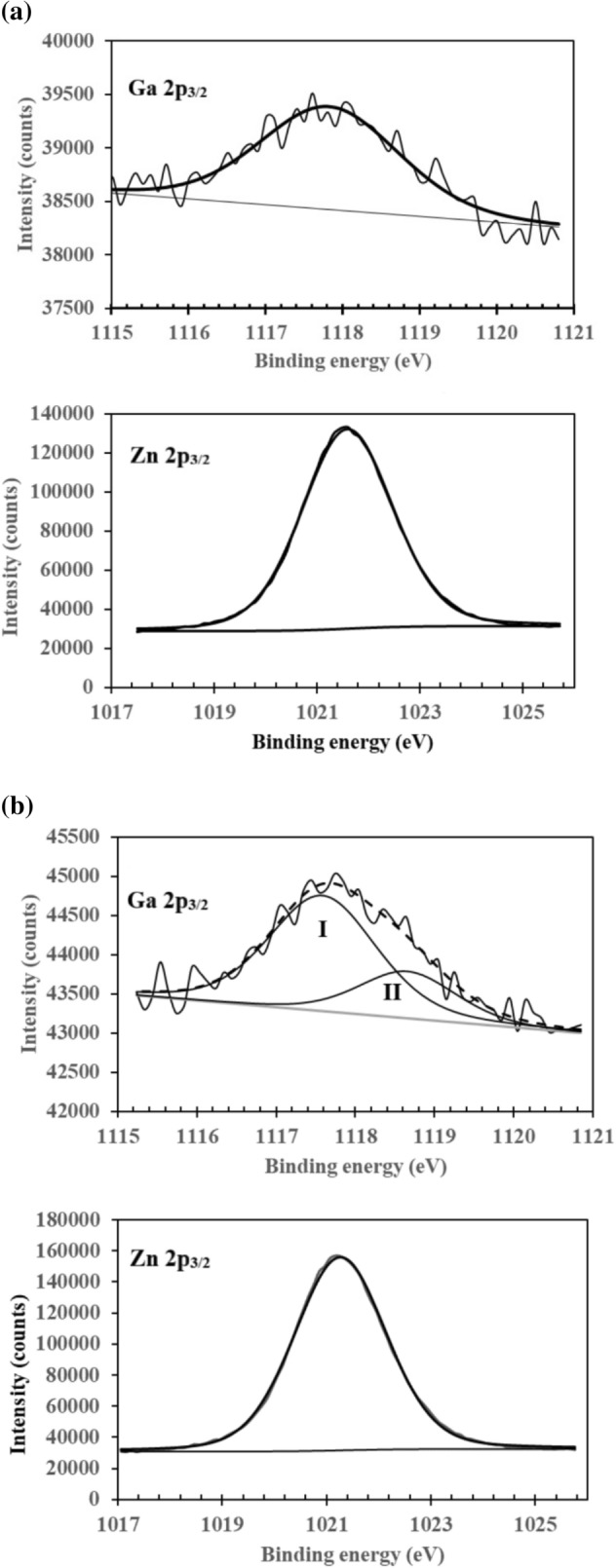
The XPS Ga 2p_3/2_ and Zn 2p_3/2_ spectra of the (**a**) 1 wt% and (**b**) 7 wt% GZO.

Here Ga_2_O_3_ represents the trivalent metal oxide used as the source of dopant in the preparation of the ZnO sputtering target. Equation (1) can be conceptually simplified to

2 $${\text{M}}_{{{\text{Zn}}}} \to {\text{M}}_{{{\text{Zn}}}}^{ \cdot } + e^{\prime}$$

where M is a trivalent metal. In GZO, Ga acts as a donor impurity, occupies the cation sites in the ZnO host lattice and releases an electron to the conduction band according to Eq. (2). The electron is only loosely bound, and thermal ionization is sufficient to cause it to enter into the conduction band^37^. Evidence of the Zn^2+^ ion in the ZnO host lattice replaced by Ga^3+^ is found in previous studies using time-differential perturbed angular correlation^38^ and nuclear magnetic resonance^39^. The XPS regional analysis indicates that Ga exists in a single chemical state for the 1 wt% film but exists in two states for the 7 wt% film. A single component Ga 2p_3/2_ peak is observed at 1117.82 eV (FWHM = 2.12 eV) for the 1 wt% GZO film (Fig. 6a), which is assigned to the Ga^3+^ ions substituting the Zn^2+^ ions in the ZnO host lattice^12,27,40,41^. For the 7 wt% film, the deconvolution of the Ga 2p_3/2_ spectrum reveals the presence of two peaks at 1117.59 eV (FWHM = 1.60 eV) (peak I) and 1118.66 eV (FWHM = 1.44 eV) (peak II) (Fig. 6b). The latter peak is attributed to Ga–O bonding from the formation of Ga‒O clusters such as GaO_x_ suboxides and oxides due to the intragrain congregation and grain-boundary segregation^23,40^. The limit of Ga solubility in ZnO is of the order of 10^21^ cm^‒3^^41^. Excess Ga has been known to segregate at grain boundaries in GZO films sputtered from ZnO targets with Ga_2_O_3_ content exceeding 5 wt%^41^. The presence of similar within-the-rule and out-of-the-rule ion fragments in the 1 and 7 wt% films indicates that excess Ga does not affect the fragmentation behaviour. A single component of Zn 2p_3/2_ peak is observed for both the 1 and 7 wt% films at binding energies 1021.59 eV (FWHM = 2.08 eV) and 1021.27 eV (FWHM = 2.08 eV), respectively. The core level binding energy of the Zn 2p_3/2_ peak in both GZO films implies that Zn atoms are in the + 2 oxidation state^12,25^. An increase in Ga doping as in the 7 wt% GZO film does not affect the Zn 2p peak^40^.

## Conclusion

ToF–SIMS fragment analysis of the GZO and undoped ZnO thin films suggests that the FAR rule applies only to the negative secondary ion fragments where within-the-rule ZnO^–^, ^66^ZnO^‒^, ^68^ZnO^–^ and ZnO_2_^‒^ ion fragments appear. In the positive secondary ion spectrum, out-of-the-rule ZnO^+^, ^66^ZnO^+^and ^68^ZnO^+^ are observed. In the GZO films, the Ga ion peaks are more intense in the positive spectrum than in the negative spectrum. The results imply that the substitution of Zn^2+^ ions by Ga^3+^ ions does not affect the fragmentation behaviour. XPS analysis of the Ga 2p_3/2_ core region indicates the presence of excess Ga above the ZnO lattice limit of solubility in the 7 wt% film but this condition also does not affect the fragmentation behaviour.

## Materials and methods

The GZO thin films were prepared using radio frequency magnetron sputtering. The sputtering targets to prepare the GZO thin films were made from a mixture consisting of ZnO and the desired amounts of Ga_2_O_3_ which were then pressed and sintered at 900 °C for 3 h. Targets with different weight percentages (1 and 7 wt%) of Ga_2_O_3_ were used in this work. For instance, the 1 wt% GZO target was fabricated using 99 g of ZnO for every 1 g of Ga_2_O_3_, and subsequently sintered. The target was attached to a Cu back plate for support and cooling to prevent it from cracking due to possible overheating during the sputtering process. The Si (100) substrates were cleaned in acetone, ethanol and methanol in an ultrasonic bath sequentially for 5 min. A base pressure of 10^–5^ Torr was achieved before the sputtering process was performed in a pure Ar gas environment at the substrate temperature of 150 °C. The Ar flow rate was maintained at 13 sccm. The native oxide layer on the Si substrate was not removed. The thickness of the films was 450–650 nm. The deposition rate was about 7 nm/min. Evidence of Ga in the 1 and 7 wt% films was ascertained by XPS using a monochromated Al Kα radiation (*hν* = 1486.6 eV) as the excitation source and an analyzer pass energy of 280 eV. Surface morphology images of the films were taken using FE-SEM while the crystallographic orientation was determined using XRD with a fixed copper anode operating at 40 kV and 30 mA. The FE-SEM images were obtained using the Leo Supra 50 VP instrument. The XRD spectra were obtained with PANalytical X’Pert Pro instrument using Cu Kα radiation (λ = 0.1541 nm). Raman measurements were performed on a Renishaw RM1000 micro-Raman spectrometer using the 514.5 nm argon ion laser. The measurements were performed with 10 mW of laser power. All Raman spectra were taken in the backscattering configuration at room temperature.

ToF–SIMS measurements were performed on the ToF–SIMS V instrument with a reflectron time-of-flight analyser for high secondary ion transmission. The spectra were obtained using Bi_1_^+^ as the primary ion beam. The primary ion beam energy was 30 keV while the pulse width was 10 ns and the cycle time 100 μs. The primary ion dose density is ~ 3 × 10^11^ ions/cm^2^, which is below the static SIMS limit. The primary ion beam current was 0.94 pA for a 3 min analysis duration. Mass spectra were obtained in bunch mode with moderate primary ion current at high mass resolution (*m*/Δ*m*) of 12,100 at *m/z* = 29 after pre-sputtering (for surface cleaning) for 5*s* using separate O_2_^+^ and Cs^+^ sources for positive and negative spectra acquisition, respectively. An electron flood source was used for charge neutralization.

Depth profiling was performed in the dual beam mode. An oxygen sputter gun operated at 1 keV and 294.30 nA was used for depth profiling in the positive polarity while Cs (1 keV, 78.70 nA) was used in the negative polarity. The sputter area was 300 μm × 300 μm while the area of analysis was 100 μm × 100 μm. The primary Bi_1_^+^ beam energy was maintained at 30 keV with a beam current of 1 pA. The pulse width was 20 ns and cycle time 30 μs. The analysis base pressure of the system was 8.5 × 10^‒11^ mbar. The chemical state of Ga was investigated using high resolution XPS measurements. The analyzer pass energy was 112 eV. The C 1*s* peak of adventitious carbon at 284.75 eV was used for charge referencing. The XPS measurements were obtained using the ULVAC-PHI Quantera II instrument.

## Supplementary Information

Supplementary Information
