# Supplementary material for: Time-of-flight secondary ion mass spectrometry fragment regularity in gallium-doped zinc oxide thin films
Source: Sci Rep. 2021 Apr 7;11:7644. doi: 10.1038/s41598-021-87386-6 (PMC8027856; doi:10.1038/s41598-021-87386-6)
Supplement: Supplementary file 1 — Supplementary Information [file 41598_2021_87386_MOESM1_ESM.pdf]

SUPPLEMENTARY INFORMATION

Time-of-flight secondary ion mass spectrometry fragment regularity in gallium-doped zinc oxide thin films

K.G. Saw[1, a)], S. R. Esa[2, b)]

[1]*School of Distance Education, Universiti Sains Malaysia, 11800 Penang, Malaysia.*
[2]*MIMOS Semiconductor (M) Sdn Bhd, Technology Park Malaysia, 57000 Malaysia.*

[a)]*Corresponding author: kgsaw@usm.my*
[b)]*rahmah.esa@mimos.my*

**Assignment**	***m/z***	**Corrected Intensity**
CH_2	14.015	32365
CH_3	15.023	124348
C_2H_2	26.016	75304
C_2H_3	27.024	844405
C_2H_4	28.031	113371
C_2H_5	29.039	776096
C_2H_6	30.043	18270
C_3H_3	39.024	408240
C_3H_5	41.039	762793
C_3H_6	42.047	117451
C_3H_7	43.056	634242
C_2H_4O	44.027	2369
C_4H_5	53.040	139818
C_4H_6	54.049	86103
C_4H_7	55.057	424010
C_4H_9	57.073	182595
^{64}Zn	63.930	294444
^{64}ZnH	64.938	140186
^{66}Zn	65.927	160863
C_5H_7	67.057	132057
^{68}Zn	67.927	113971
C_5H_9	69.074	102796
^{64}ZnO	79.924	752
C_6H_9	81.075	59460
^{66}ZnO	81.922	275

Supplementary Table 1. Positive ion fragments of undoped ZnO film corresponding to Supplementary Figure 1.

Assignment	*m/z*	Corrected Intensity
C	12.000	256883
CH	13.008	1287666
CH_2	14.016	308566
O	15.995	1650108
OH	17.002	2972406
C_2	24.000	186331
C_2H	25.008	858175
CN	26.003	27651
C_3H_2	38.017	54705
C_2HO	41.004	59147
C_3H_5	41.043	6383
CHO_2	44.999	73063
C_4H_3	51.025	28050
$C_2H_2O_2$	58.008	57411
$C_2H_3O_2$	59.017	21985
$C_3H_3O_2$	71.018	94130
^{64}ZnO	79.924	19982
$^{64}ZnOH$	80.933	40502
^{66}ZnO	81.921	11388
$^{66}ZnOH$	82.930	24570
^{68}ZnO	83.922	10743
$^{68}ZnOH$	84.929	16184
$^{64}ZnO_2$	95.920	3088
$^{64}ZnO_2H$	96.929	11909

Supplementary Table 2. Negative ion fragments of undoped ZnO film corresponding to Supplementary Figure 2.

Sample	C (at%)	O (at%)	Zn (at%)
Undoped ZnO film	1.20	42.30	56.50

Supplementary Table 3. XPS survey analysis of undoped ZnO film.

Sample	C (wt%)	O (wt%)	Zn (wt%)	Ga (wt%)
1 wt% GZO film	1.22	18.01	79.93	0.83
7 wt% GZO film	4.22	23.40	67.21	5.17

Note: The 1% wt GZO target was fabricated using 99 g of ZnO for every 1 g of Ga_2O_3. 1 g of Ga_2O_3 contains 0.74 g Ga. Similarly, 7 g of Ga_2O_3 contains 5.20 g Ga. In the wide survey scans of the GZO thin films, XPS quantification uncertainties can be ± 10% of the measured value.

Supplementary Table 4. XPS survey analysis of 1 and 7 wt% GZO films.

Assignment	*m/z*	Corrected Intensity
H	1.007	48846
C	11.999	5803
O	15.994	6809
OH	17.002	6835
Na	22.989	117165
Si	27.976	31426
K	38.964	54615
C_3H_5	41.040	17839
C_3H_7	43.056	8379
^{64}Zn	63.928	366284
^{66}Zn	65.925	197838
^{68}Zn	67.924	127896
Ga	68.925	175283
^{71}Ga	70.924	111906
ZnO	79.922	7399
ZnOH	80.930	80824
^{66}ZnO	81.919	3778
^{68}ZnO	83.933	25033
GaO	84.926	31249
GaOH	85.934	10618

Supplementary Table 5. Positive ion fragments of 1 wt% GZO film corresponding to Figure 2a.

Assignment	*m/z*	Corrected Intensity
H	1.007	48846
C	11.999	5803
CH_3	15.023	12383
Na	22.990	471755
Si	27.976	6823
K	38.963	96757
C_3H_5	41.040	54033
C_3H_7	43.056	29058
^{64}Zn	63.928	191815
^{66}Zn	65.925	103728
Ga	68.925	784845
^{71}Ga	70.924	516188
^{64}ZnO	79.922	3837
$^{64}ZnOH$	80.931	39907
^{66}ZnO	81.919	1919
^{68}ZnO	83.934	14403
GaO	84.926	16311
GaOH	85.931	10779
GaH_2O	86.934	7727

Supplementary Table 6. Positive ion fragments of 7 wt% GZO film corresponding to Figure 2b.

Assignment	*m/z*	Corrected Intensity
H	1.010	5936302
C	12.004	2142159
CH	13.011	2021419
O	15.998	7059203
OH	17.006	6901020
C_2	24.004	6826028
C_2H	25.002	6810463
S	31.973	702941
Cl	34.969	171965
^{37}Cl	36.966	53106
C_2O	39.996	1822461
CNO	41.999	291228
CHO_2	44.998	932772
CH_2OF	49.010	1954256
SiO_2	59.965	824047
SiH_2O	60.971	261158
^{64}Zn	63.941	1538
Ga	68.927	7827
^{71}Ga	70.925	826
C_6	71.997	327358
C_6H	73.006	511165
^{64}ZnO	79.918	1337373
$^{64}ZnOH$	80.926	1389812
^{66}ZnO	81.913	765150
^{68}ZnO	83.913	621618
GaO	84.919	641429
$^{64}ZnO_2$	95.910	361644
COGa	96.919	859679
H_2SiGa	98.915	537392

Supplementary Table 7. Negative ion fragments of 1 wt% GZO film corresponding to Figure 4a.

**Assignment**	***m/z***	**Corrected Intensity**
H	1.009	4574035
C	12.002	1238985
CH	13.010	1687252
O	15.997	6945203
OH	17.005	6832535
C_2	24.003	4480514
C_2H	25.011	5445635
S	31.972	196952
Cl	34.969	207398
^{37}Cl	36.966	64618
C_2O	39.996	1090482
CNO	42.000	501558
CHO_2	44.999	791761
CH_2OF	49.010	692479
SiO_2	59.967	506632
SiH_2O	60.974	166254
^{64}Zn	63.943	178
Ga	68.929	8394
^{71}Ga	70.927	2472
^{64}ZnO	79.922	1064293
$^{64}ZnOH$	80.931	1222807
^{66}ZnO	81.918	606917
^{68}ZnO	83.919	503131
GaO	84.922	1219193
$^{64}ZnO_2$	95.917	335701
COGa	96.926	875796
H_2SiGa	98.922	552021

Supplementary Table 8. Negative ion fragments of 7 wt% GZO film corresponding to Figure 4b.

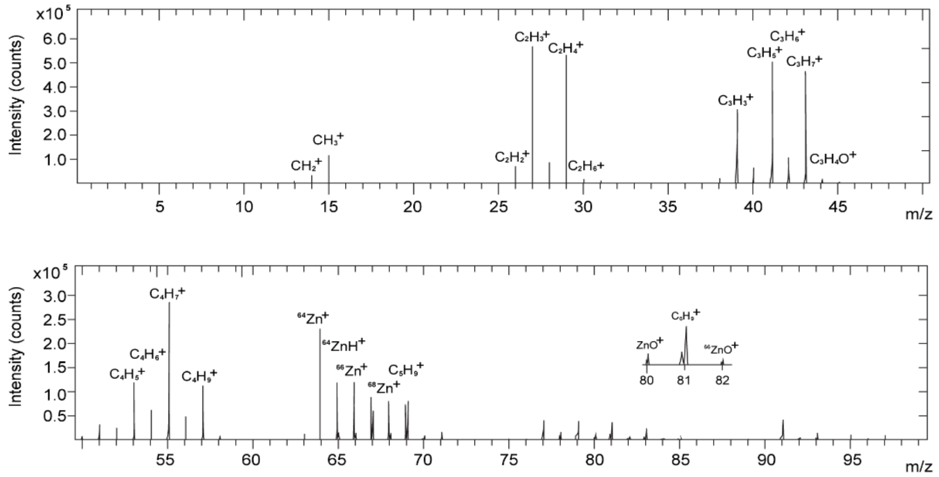

Supplementary Figure 1. Positive secondary ion spectrum of the undoped ZnO film.

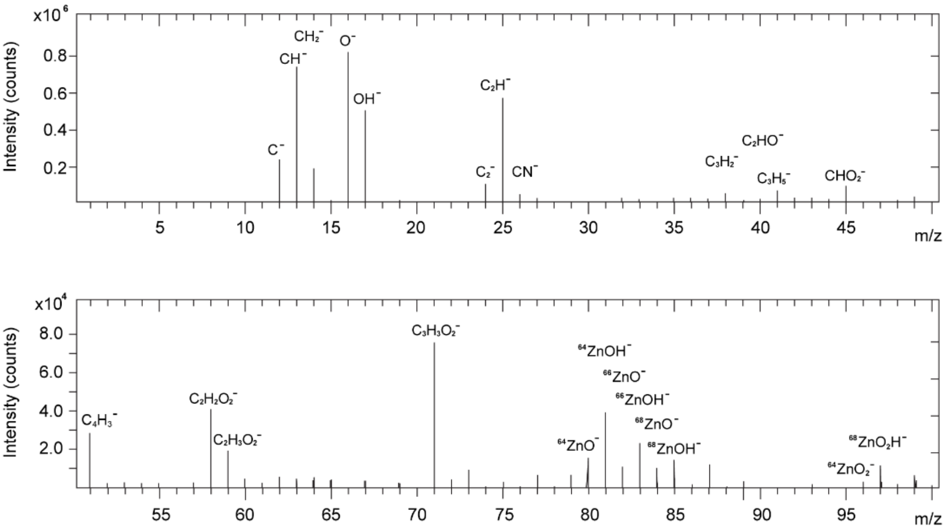

Supplementary Figure 2. Negative secondary ion spectrum of the undoped ZnO film.

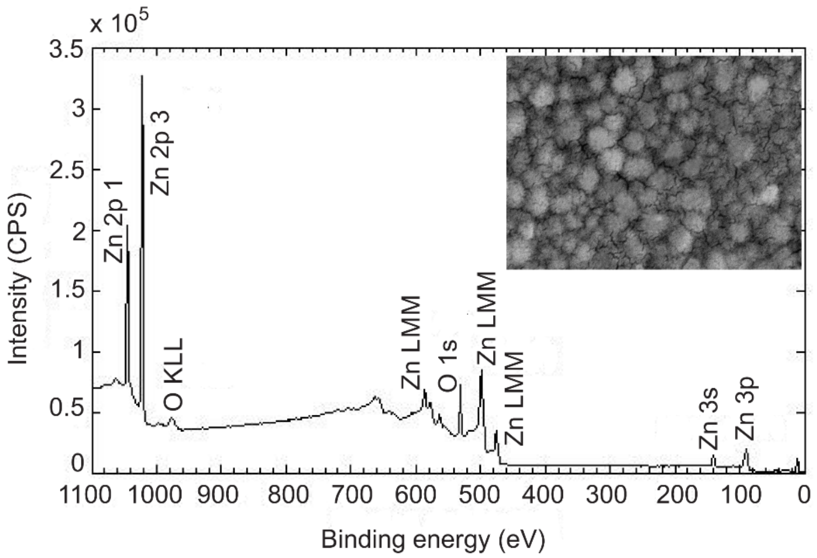

Supplementary Figure 3. XPS survey spectrum of the undoped ZnO film. Inset is the FE-SEM image showing a continuous surface morphology (mag. 51000X).

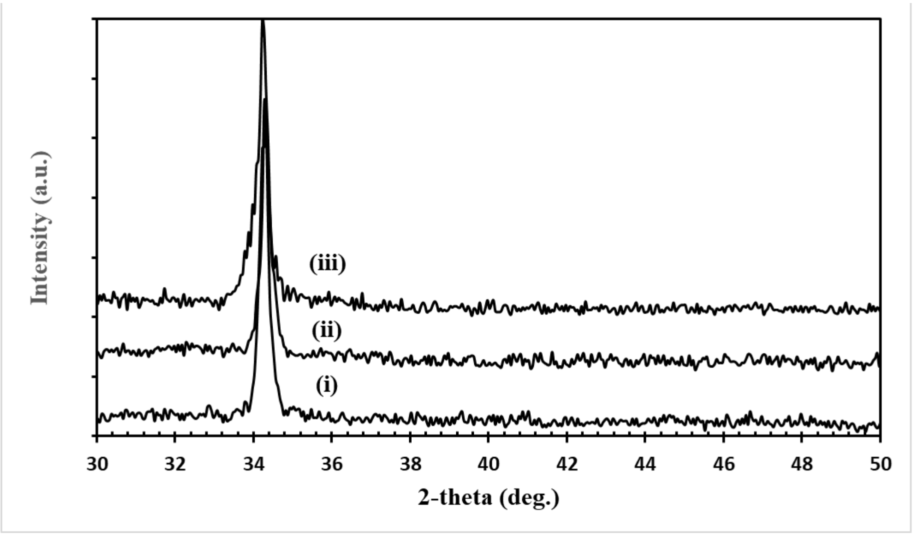

Supplementary Figure 4. XRD 2-theta spectra of the 1 and 7 wt% GZO films (i) 1 wt% GZO film deposited at room temperature; (ii) 1 wt% GZO film deposited at 150°C; (iii) 7 wt% GZO film deposited at 150°C.

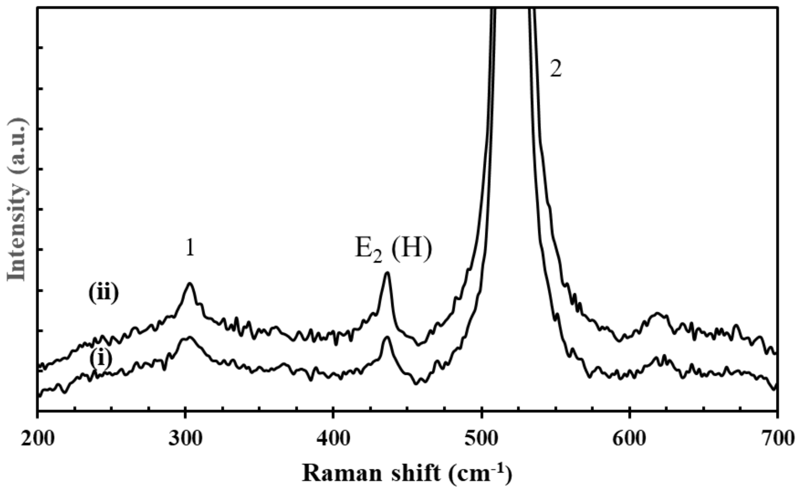

Supplementary Figure 5. Raman spectra of the 1 and 7 wt% GZO films (i) 1 wt% GZO film; (ii) 7 wt% GZO film.
